# Clinical and economic impact of a TEE monitoring system in intensive care

**DOI:** 10.1186/cc9447

**Published:** 2011-03-11

**Authors:** HM Hastings, SL Roth

**Affiliations:** 1 ImaCor, Garden City, NY, USA

## Introduction

The purpose of this study was to determine the clinical and economic impact of hemodynamic monitoring in intensive care with the ImaCor TEE monitoring system, including a miniaturized, detachable, single-use probe (the ImaCor ClariTEE™). TEE has been cited as especially appropriate for hemodynamic monitoring because abnormalities are multifactorial; or example, hypovolemia, LV and RV dysfunction, tamponade. Unlike conventional probes, the ClariTEE™ was designed and cleared by the FDA to remain indwelling for 72 hours of episodic hemodynamic monitoring.

## Methods

The ImaCor system was used to monitor 46 postcardiac surgery patients at two institutions and 68 general ICU patients at eight institutions. Effects on management were recorded and analyzed retrospectively. Economic impact was estimated from [1-4].

## Results

In 46 postcardiac surgery patients, surgical re-exploration was avoided in five patients (11%), and fluid and pressor administration changed in 23 patients (50%). TEE monitoring also detected tamponade requiring reoperation and helped optimize the LVAD flow rate. Even without including likely reductions in acute kidney injury, a common complication [5], estimated hospital charges (see [1-4]) were reduced by $12,000 per patient. In 68 general ICU patients, fluid and pressor administration was changed in 28 patients (41%), reducing estimated hospital charges by $7,400 per patient.

## Conclusions

TEE monitoring demonstrated the potential to improve hemodynamic management; expected to reduce hospital stay [6,7]: even small amounts of mild instability significantly increase hospital stay and charges [4]. TEE monitoring also demonstrated the potential to avoid reoperation postcardiac surgery. Reoperation significantly increases morbidity (low cardiac output, acute renal failure, sepsis), vent time, ICU stay and mortality [8]; also cost [1]. Although further study is needed, TEE monitoring has shown potential for significant clinical and economic impact.
